# Cisplatin-Resistance in Oral Squamous Cell Carcinoma: Regulation by Tumor Cell-Derived Extracellular Vesicles

**DOI:** 10.3390/cancers11081166

**Published:** 2019-08-14

**Authors:** Xin-Hui Khoo, Ian C. Paterson, Bey-Hing Goh, Wai-Leng Lee

**Affiliations:** 1 School of Science, Monash University Malaysia, Subang Jaya 47500, Selangor, Malaysia; 2 Department of Oral and Craniofacial Sciences, University Malaya, Kuala Lumpur 50603, Malaysia; 3 Biofunctional Molecule Exploratory Research Group (BMEX), School of Pharmacy, Monash University Malaysia, Subang Jaya 47500, Selangor, Malaysia; 4 Health and Well-being Cluster, Global Asia in the 21st Century (GA21) Platform, Monash University Malaysia, Subang Jaya 47500, Selangor, Malaysia

**Keywords:** oral squamous cell carcinoma, extracellular vesicles, cisplatin, drug resistance, protein profiling

## Abstract

Drug resistance remains a severe problem in most chemotherapy regimes. Recently, it has been suggested that cancer cell-derived extracellular vesicles (EVs) could mediate drug resistance. In this study, the role of EVs in mediating the response of oral squamous cell carcinoma (OSCC) cells to cisplatin was investigated. We isolated and characterized EVs from OSCC cell lines showing differential sensitivities to cisplatin. Increased EV production was observed in both de novo (H314) and adaptive (H103/cisD2) resistant lines compared to sensitive H103 cells. The protein profiles of these EVs were then analyzed. Differences in the proteome of EVs secreted by H103 and H103/cisD2 indicated that adaptation to cisplatin treatment caused significant changes in the secreted nanovesicles. Intriguingly, both resistant H103/cisD2 and H314 cells shared a highly similar EV protein profile including downregulation of the metal ion transporter, ATP1B3, in the EVs implicating altered drug delivery. ICP-MS analysis revealed that less cisplatin accumulated in the resistant cells, but higher levels were detected in their EVs. Therefore, we inhibited EV secretion from the cells using a proton pump inhibitor and observed an increased drug sensitivity in cisplatin-resistant H314 cells. This finding suggests that control of EV secretion could be a potential strategy to enhance the efficacy of cancer treatment.

## 1. Introduction

Oral cancer is ranked the 16th most common form of cancer and accounted for 2% of all newly diagnosed cancer cases in 2018 [1]. Up to 90% of oral malignancies are diagnosed as oral squamous cell carcinoma (OSCC) [2]. Chemotherapy as an optional combination treatment for OSCC, has been shown to enhance the overall survival of patients with advanced disease. However, the emergence of drug resistance becomes a major challenge for OSCC patients undergoing chemotherapy [2,3]. There are two main types of drug resistance described in cancers, namely, de novo or intrinsic drug resistance where the cells are inherently insensitive to chemotherapeutic drugs, and acquired or adaptive drug resistance that develops over time after prolonged exposure to chemotherapeutic drugs [4]. Various mechanisms of drug resistance have been identified [5] and recently, extracellular vesicles have been identified as a key regulator of drug resistance in many cancer types.

The term “extracellular vesicle” (EV) is generally used to describe the particle released naturally from a cell. These particles are spherical vesicles containing a lipid bilayer but lack a functional nucleus. Therefore, EVs are unable to replicate [6]. These nanovesicles play roles in the removal of unwanted cellular material and in intercellular communication via the transfer of bioactive molecules packaged within the EVs [7]. To date, there have been very limited studies regarding the role of EVs in influencing the cellular response to chemotherapeutic drugs in OSCC. The only report to date showed that incubation of exosomes, a type of EVs derived from cisplatin-resistant OSCC cells could attenuate the response of recipient cells to the drug via transfer of miR-21 [8]. Furthermore, exosomes have been found to regulate drug efflux from cancer cells of various origin [9] but there are no reports of this phenomenon in oral cancer.

The extracellular pH in OSCC tumor is considerably more acidic than in normal tissue. Vacuolar ATPases(V-ATPases), which is responsible for regulating acidity in the microenvironment of solid tumor could be the important molecule in regulating the drug resistance mechanisms in such a tumor. The use of specific V-ATPase inhibitors, the proton pump inhibitors (PPIs) was found to sensitize tumor cell lines treated with different chemotherapy drugs, thus could be a possible means of controlling resistance to antitumor drugs [10]. On the other hand, reduction of exosomes spill-over into blood stream using pretreatment of PPI had been reported to retain more cisplatin in nude mice bearing human melanoma tumors [11]. While, the lower levels of plasmatic exosomes were shown to correlate with a better life expectancy of OSCC patients [12]. These studies have suggested possible correlation between EVs and V-ATPase, which could be a novel strategy to control drug resistance in OSCC for a better treatment outcome.

In this study, we have used OSCC cell lines that display de novo and acquired resistance to cisplatin to investigate the role of EVs in regulating cellular responses to cisplatin. To explore the functional biomolecules involved in regulating the response to cisplatin, EV protein profiles from sensitive and resistant OSCC cells were compared. Our findings demonstrate that OSCC-derived EVs may regulate cisplatin resistance through a cellular efflux system and inhibition of EV release might represent a new therapeutic approach, which could sensitize OSCCs to chemotherapy.

## 2. Results

### 2.1. Screening and Establishment of Resistant Cell Lines

**Figure 1:**
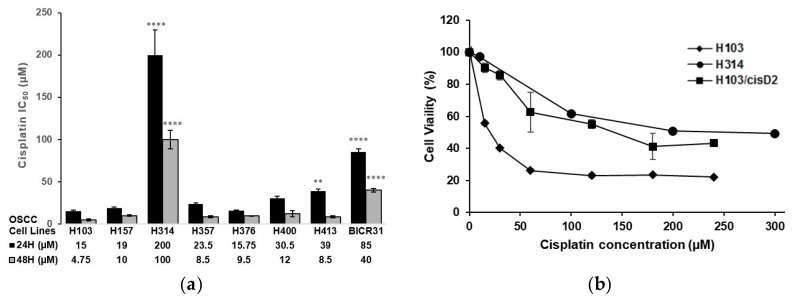
Cisplatin responses of oral squamous cell carcinoma (OSCC) cell lines. (**a**) IC_50_ of OSCC cell lines treated with cisplatin for 24 and 48 h. Data were expressed as mean of at least 4 replicates. IC_50_ with significant difference compared to H103 cell lines were indicated with asterisks (*n* = 4, ** *p* < 0.01, **** *p* < 0.0001); (**b**) Cisplatin response curve of the H103 and H103 resistant subline, H103/cisD2 and H314 for 24 h. IC_50_ for each cell line was determined by interpolating at 50% cell viability.

To select OSCC cell lines with distinct sensitivities to cisplatin, the response of eight OSCC cell lines [13,14] to the drug was determined using 3-(4,5-dimethylthiazol-2-yl)-2,5-diphenyl tetrazolium bromide (MTT) assays. Figure 1a shows the IC_50_ values of these cell lines following treatment with cisplatin for 24 and 48 h. H103 had the lowest IC_50_ (15 µM and 4.57 µM for 24 and 48 h, respectively) whilst H314 was the most resistant (IC_50_ of 200 µM and 100 µM for 24 and 48 h, respectively) (Figure 1a). After several pulse treatments with cisplatin, a 10-fold higher IC_50_ (150 µM) was observed in one of the resistant lines (H103/cisD2) derived from H103 cells. H103/cisD2 cells were morphologically different from their parental cells (Figure S1) and demonstrated a similar response to that of the de novo resistant cell line, H314, to cisplatin treatment (Figure 1b).

### 2.2. Characterization of OSCC Cell-Derived EVs

**Figure 2:**
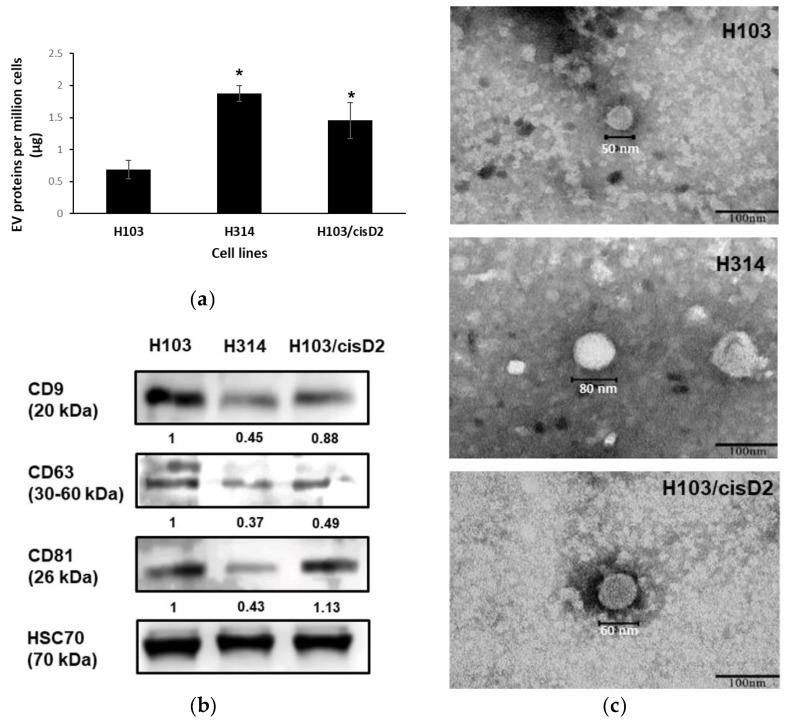
Characterization of oral squamous cell carcinoma (OSCC)-cells derive extracellular vesicles (EVs). (**a**) EVs production of OSCC cells. * indicate significant differences, *n* = 3, *p* < 0.05 (One-way Annova, Tukey’s post-hoc). (**b**) Western blot of EV marker protein expression identified in OSCC cells-derived EVs. The images are representative of three independent experiments (Figure S4). All the signals were normalized against intensity of HSC70. (**c**) TEM images of single EV derived from OSCC cell lines (scale bar: 100 nm).

EVs were isolated from H103, H314 and H103/cisD2 cell lines using differential ultracentrifugation. Protein quantification indicated that the resistant lines (H314 and H103/cisD2) produced 1.88 µg and 1.45 µg of EV protein per million cells, respectively, which is 2–2.7 fold higher than sensitive H103 cells (0.69 µg per million cells) (Figure 2a). The EV marker proteins (tetraspanins: CD9, CD63, CD81 and heat shock protein: HSC70) were detected in the isolated vesicles with a consistent and significant high level of HSC70 expressed in all the OSCC cell-derived EVs (Figure 2b). Quantitative analysis shows that sensitive H103 cells secreted EVs expressing the highest levels of all three tetraspanins, while the lowest expression was observed in those from the resistant line H314 (Figure S2). The morphology of the OSCC-derived EVs was studied using TEM. The EVs isolated from all the OSCC cells were spherical with a size range of 40–80 nm (Figure 2c). Further, particle size analysis using Nanosight demonstrated a comprehensive measurement of the size distribution of all the EVs in the preparation (Table 1). The average values [(99.8 ± 16.3)–(130.0 ± 9.7) nm] observed in this study are similar to the reported sizes of small EVs or exosomes isolated from other cell lines [6,15]. Together, these characteristics confirmed that the particles isolated from OSCC cells in this study were CD9+/CD63+/CD81+-small EVs.

### 2.3. Protein Profiling of OSCC Cell-Derived EVs

**Figure 3:**
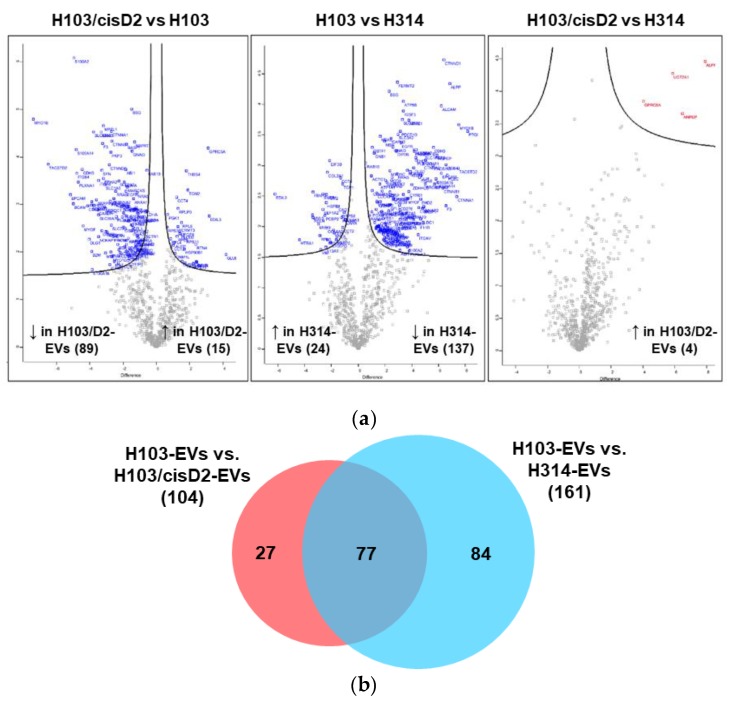

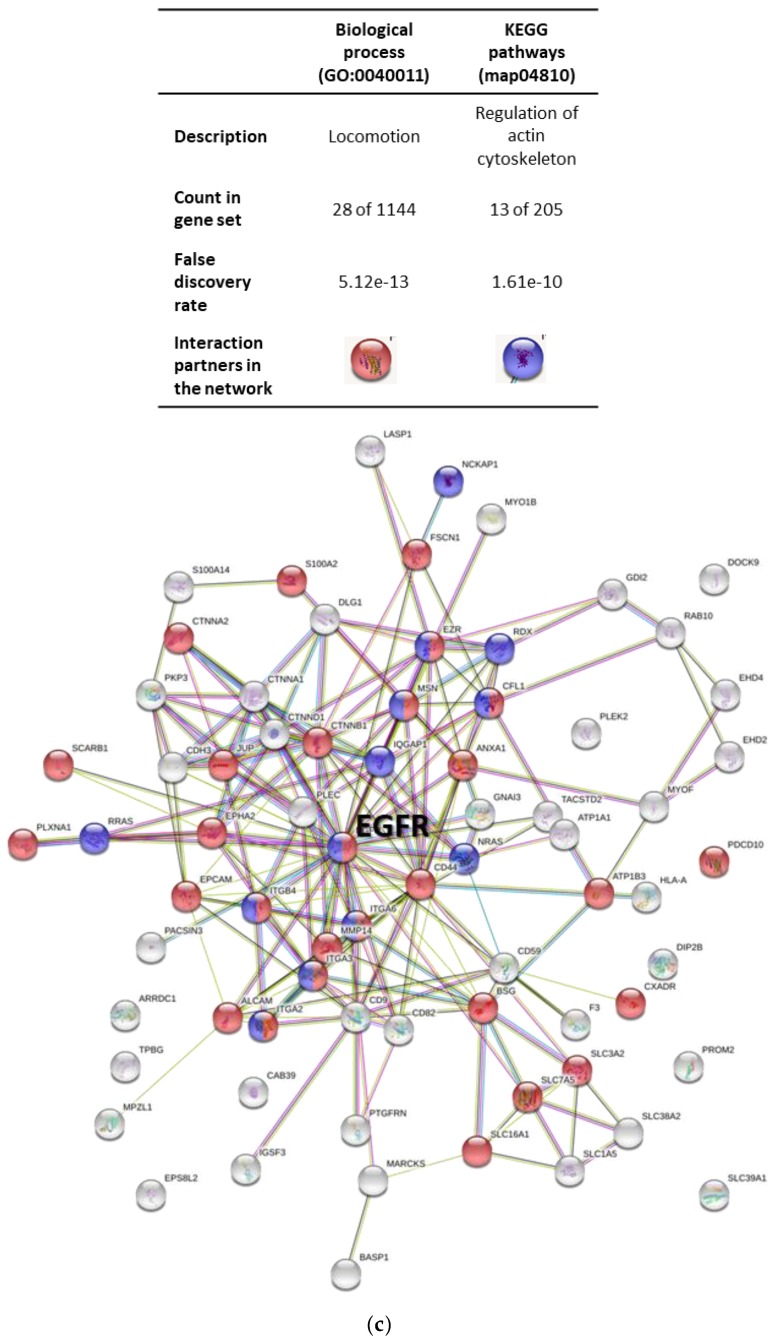
Protein profile and network of OSCC derived EVs. (**a**) Volcano scatter plots showing proteins with differential expression in the EVs derived from OSCC cells. (**b**) Venn chart depicted the overlapping of the differentially expressed proteins in EVs (all published in Vesiclepedia) derived from three OSCC cell lines. (**c**) Networks of proteins which downregulated in the EVs derived from cisplatin-resistant OSCC cells (STRING database: https://string-db.org/).

Following the profiling of proteins in the EVs isolated from H103, H314 and H103/cisD2 cells, a total of 1387 proteins were identified across all the analyzed samples falling below a predefined FDR threshold of 1%. A total of 765 proteins were then quantified successfully. When compared to the proteins identified in EVs derived from H103 cells, there were 104 proteins differentially expressed in EVs derived from H103/cisD2 cells (15 up regulated; 89 down regulated) and 161 for those from H314 cells (24 up regulated; 137 down-regulated) (Figure 3a). Despite originating from different cell lines, only four proteins were observed to be up-regulated in EVs derived from H103/cisD2 cells as compared to those of H314 cells, indicating high similarity in terms of proteins present in EVs derived from resistant cells. All the differentially expressed proteins in the EVs originating from different OSCC cell lines could be mapped to protein IDs within the Vesiclepedia using FunRich software indicating no novel EV proteins were identified in this study. Figure 3b shows a Venn chart presenting a further comparison between the differential expressed protein lists. Among those proteins differentially expressed in EVs derived from H103/cisD2 and H314 compared to those from H103 respectively, 77 were found as a common set (Table S1). Protein network analysis using STRING suggested that most of these cisplatin resistance-associated EV proteins may be involved in EGFR-associated networks (Figure 3c). Further, enrichment analyses using GO and KEGG pathway analyses showed the protein networks may function in locomotion and regulation of the actin cytoskeleton respectively. Comprehensive outcomes of the enrichment analyses are listed in Tables S2–S4.

### 2.4. Regulation of EGFR in OSCC Cell-Derived EVs

**Figure 4:**
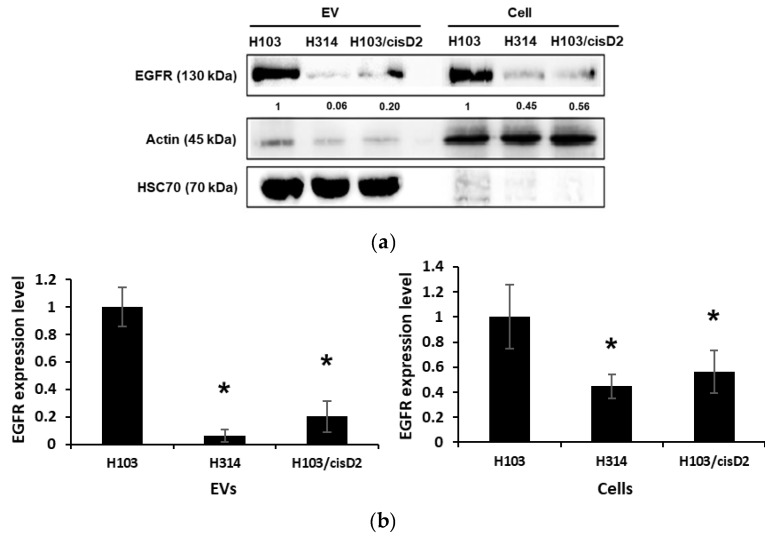
Regulation of EGFR in OSCC-derived EVs. (**a**) Western blot image of EGFR expression in OSCC cells and EVs. Signals of EV proteins were normalized against intensity of HSC70 while those of cell lysate were against actin. (**b**) Quantification of EGFR in EVs and in cells. Experiments were conducted in triplicates (Figure S4) and represented as ±SEM. * indicated significant differences, *p* < 0.05, *n* = 3 (One-way Annova, Tukey’s post-hoc).

As EGFR was identified as the hub of most of the networks connecting the differentially expressed proteins in EVs derived from resistant cells (Figure 3c), its expression in EVs and OSCC cells was verified using Western blotting (Figure 4a). In the proteomic analysis, EGFR was found to be reduced by 1.8 and 3.2-fold in the EVs secreted from H103/cisD2 and H314 cells, respectively (Table S1). Consistently, an 80–90% decrease of EGFR expression was observed in the EVs derived from the two resistant cell lines while there was approximately a 50% reduction in cell lysates (Figure 4b). EGFR is known for its function in regulating cell proliferation [16] and in agreement with the expression levels of EGFR, H314 and H103/cisD2 cells showed lower growth rates when compared to H103 cells (Table 2).

### 2.5. Regulation of Drug Resistance-Associated Proteins in OSCC Cell-Derived EVs

**Figure 5:**
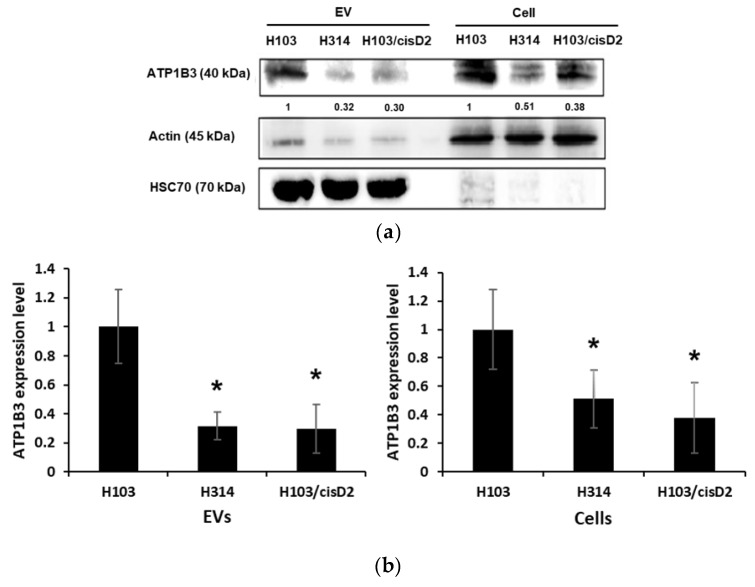

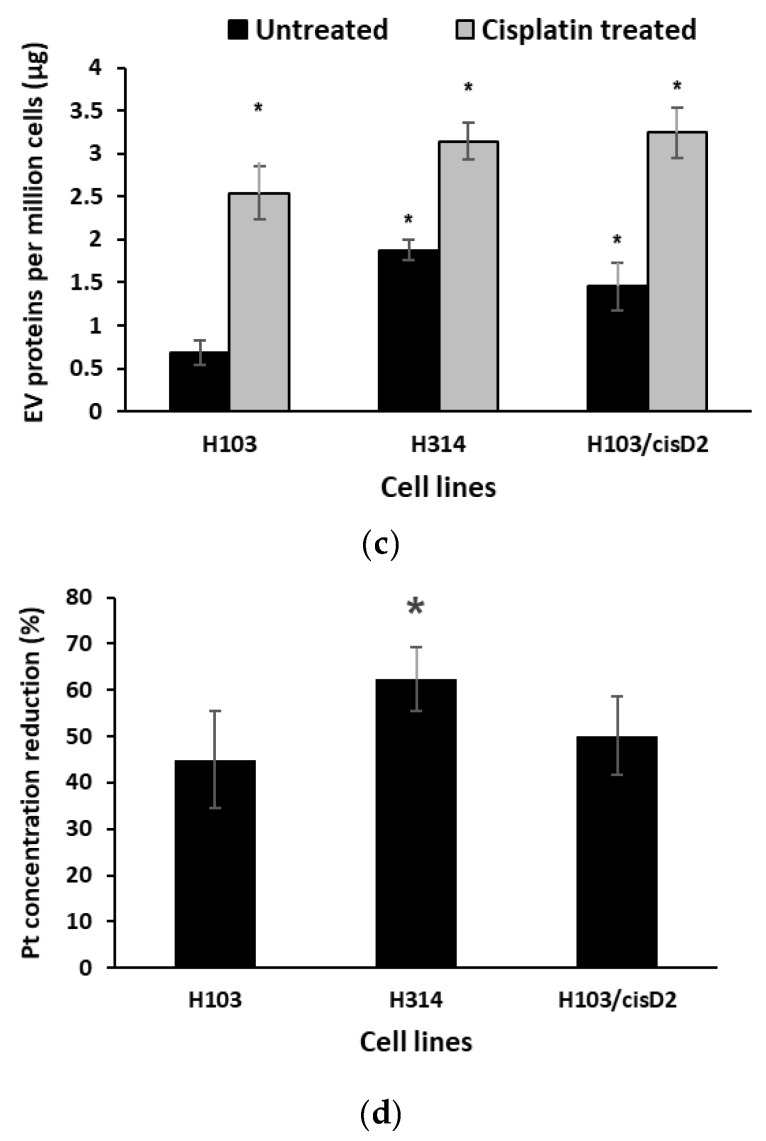
Regulation of drug resistance-associated proteins in OSCC cell-derived EVs. (**a**) Western blot images of ATP1B3 expression in OSCC cells and EVs. Signals of EV proteins were normalized against intensity of HSC70 while those of cell lysate were against actin. (**b**) Quantification of ATP1B3 expression level in EVs and cell lysates. (**c**) EVs production by OSCC cells with and without cisplatin treatment. (**d**) Percentage of cisplatin reduction in OSCC cell lysate within 10 h after treatment. * indicate significant differences, *n* = 3, *p* < 0.05 (One-way Annova, Tukey’s post-hoc).

Protein profiles of EVs secreted from both resistant H103/cisD2 and H314 lines are highly similar with 77 proteins similarly regulated when compared to those from the sensitive H103 line (1). Only four proteins were found to have higher levels in EVs from cisplatin resistant cells and those with more than two-fold change include EGF-like repeats and discoidin I-like domains 3 (EDIL3) and transglutaminase 2 (TGM2). Most of the EV proteins of resistant lines were found at lower levels when compared to H103 cells, including six proteins involved in the regulation of metal ion transportation (Table 3). Gene ontology described the network as the directed movement of metal ions with an electric charge, into, out of or within a cell and between cells via transporter or pore. The six proteins comprise PACSIN3 with function mainly in vesicle-mediated transport together with other proteins involved in sodium or potassium ion transportation. ATP1A1 and ATP1B3 especially are the key proteins that form the ion pump Na^+^/K^+^ ATPase that is responsible for the transportation of sodium and potassium ions [17]. Western blotting verified that the EVs derived from H314 and H103/cisD2 contain less ATP1B3 and lower levels of the protein were also observed in the resistant OSCC cells (Figure 5a,b). Downregulation of proteins modulating metal ions transportation may cause aberrant drug accumulation in the cells. We observed that both cisplatin-resistant cells produced two to three-fold higher levels of EVs when compared to those of sensitive H103 cells. When treated with cisplatin, there was an increase of EV production in all three cell lines. Cisplatin triggered almost four times more EV release from H103 cells but this was significantly lower than those from treated resistant OSCC cells (Figure 5c). Concurrently, cisplatin accumulation in treated cells was quantified by measuring intracellular platinum after 2 h of treatment followed by 10 h of incubation in drug-free medium (Figure S3). The rate of intracellular cisplatin reduction for each OSCC line was derived and is shown in Figure 5d. After 10 h in cisplatin free media, almost half of the initial cisplatin was removed from H103 cells and its resistant H103/cisD2 line while the highest rate of removal was observed in the *de novo* resistant H314 cells (62.4 ± 6.8%) (Figure 5d).

### 2.6. Effect of EV Inhibition on Drug Response of OSCC Cells

**Figure 6:**
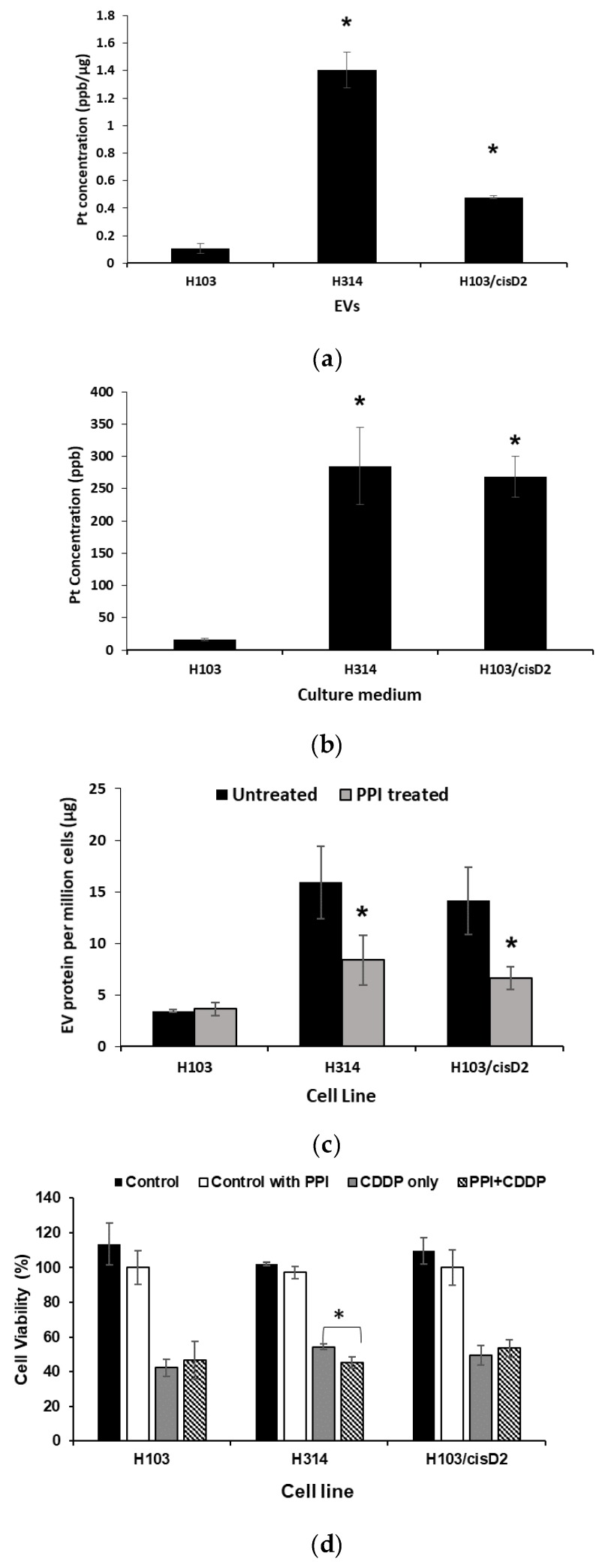
Effect of EV inhibition on drug response of OSCC cells. (**a**) Platinum concentration in EVs derived from OSCC cells. (**b**) Platinum concentration in culture medium after removal of EVs. (**c**) Inhibition of EVs produced by OSCC cell lines using PPI. (**d**) Effect of PPI treatment on cisplatin sensitivity of OSCC cells. * Indicates significant difference, *p* < 0.05, *n* = 3 (Student’s *t*-test, Tukey’s post-hoc).

To further validate the role of EVs in mediating drug resistance in OSCC cells through active drug transportation, the cisplatin content in EVs and free cisplatin in culture medium was examined by quantification of platinum levels using ICP-MS (Figure 6a,b). EVs and culture media of both resistant lines H103/cisD2 and H314 contained significantly higher levels of cisplatin when compared to the sensitive H103 line. It is worth noting that the cisplatin level in the culture media was similar (265 ppb and 285 ppb) for both resistant lines, but the drug contained in H314-derived EVs (~1.40 ppb/µg of EV protein) was 3 fold higher than those in EVs secreted from H103/cisD2 cells (~0.47 ppb/µg of EV protein) (Figure 6a,b). In line with the highest rate of cisplatin removal from H314 cells (Figure 5d), the high levels of the compound found in EVs released from the resistant cells implies that inhibition of EV secretion may reduce the efflux of cisplatin. The pretreatment of cells with 25 µM of the PPI, Lansoprazole, for 24 h could significantly inhibit EV production by H103/cisD2 (~40% reduction) and H314 cells (~60% reduction) (Figure 6c). Further, the inhibition of EV production further decreased the viability of H314 cells following cisplatin treatment, but no effect was observed in other PPI-pretreated OSCC cells (Figure 6d). Together these results demonstrate that the de novo resistant OSCC cells that secreted EVs containing high levels of cisplatin could be sensitized to the drug by inhibition of EV production.

## 3. Discussion

The heterogeneity of genotypes and phenotypes within cancer cell populations is likely to be the main factor responsible for chemotherapeutic drug resistance. In the present study, a representative panel of OSCC cell lines that were derived from oral cancer patients with various clinicopathological characteristics [13,14] was examined and showed different levels of sensitivity to cisplatin. Pulse treatment of the sensitive line, H103, with cisplatin caused an increase in drug resistance, which might reflect the clinical situation of developing drug resistance after prolonged exposure and treatment cycles of chemotherapy [18]. A resistant line H103/cisD2 was therefore developed to serve as an experimental model of adaptive resistance, while H314 cells—which showed an innate resistance phenotype was used as a model of de novo resistance. After ultracentrifugation, the nanovesicles isolated from these OSCC cells were then characterized and showed typical morphology and markers of small EVs. The protein markers such as transmembrane tetraspanins CD9, CD63, CD81 and heat shock proteins HSC70 were used in this study to perform identity verification of the isolated vesicles and they were found to share the same characteristics as small EVs including exosomes derived from many cell types [19,20]. 

Despite originating from the isogenic cell lines, the significant difference in the EV protein expression of H103 and H103/cisD2 implies that adaptation to cisplatin treatment may cause changes in the protein profiles of the secreted EVs. Intriguingly, H103/cisD2 and H314 cells shared a highly similar EV protein profile with up to 73 proteins being down regulated in these resistant cells-derived EVs. Protein network analysis illustrated that 30% of these proteins were involved in the process of cell motility and nearly half of them, including EGFR, are part of the pathway regulating the actin cytoskeleton. This echoes a recent study, which reported that the activation of the EGFR pathway could induce reorganization of cytoskeletal networks in modulating cellular behaviors, such as cell division [21]. In line with this finding, we found both resistant lines grew slower than sensitive H103 cells and the slow growing resistant cells secreted EVs with lower levels of EGFR and beta actin. Characterization of the H-series OSCC cells showed less EGFR expression in H314 which originated from stage II tumor with metastasis nodes when compared to H103 which was derived from a stage I tumor [13]. Decreased EGFR expression was also detected in invasive OSCC cells [22]. Together these findings suggest that the loss of EGFR in both cells and secreted EVs may correlate with malignant phenotypes, including invasiveness and drug resistance of OSCC cells. 

From the protein profiles, only four proteins were identified to be present at higher levels in the EVs secreted by both resistant lines compared to sensitive cells. These proteins have been shown to play important roles in cancers. For example, EDIL3 is involved in the progression of breast and bladder cancers [23,24]), while TGM2 mediates chemoresistance of both lymphoma and breast cancer [25,26]. In addition, TGM2 was found to play a role in selecting and packing of EV cargo under stressful cellular conditions [27]. The high levels of TGM2 identified in the EVs of both H314 and H103/cisD2 may imply a correlation between the resistant phenotypes of these cell lines to their EVs containing a similar proteome. As cisplatin is a metal-based compound, it is perhaps not surprising that a group of six proteins involved in metal ion transportation were found in the protein profiles of resistant cells. These proteins included Na+, K+-ATPases which were reported to facilitate the active transport of cisplatin in an OSCC cell line H4-II-E and a decreased level of this transporter protein was observed in a resistant variant [28]. Similarly, we observed lower levels of beta 3 subunit of the ATPase (ATPB3) in the resistant cells as well as their EVs when compared to those from sensitive cells, which could possibly generate a molecular setting that halts the active drug uptake by resistant OSCC cells. 

Recently, EVs containing anti-cancer drugs were observed to reduce drug accumulation in breast cancer cells [29]. In our study, cisplatin treatment was shown to stimulate higher levels of EV released from all three OSCC cell lines, while there was a more rapid decline in cellular drug levels of resistant cells. In response to a similar dose of cisplatin, high levels of the drug were detected initially in the de novo resistant H314 cells followed by a significant drop of intracellular drug levels when the cells were cultured in drug free medium. In parallel, H314 released EVs containing high levels of cisplatin. In contrast, less cisplatin accumulated in H103/cisD2 cells throughout the treatment and relatively lower doses of the drug were detected in their EVs, implying removal of intracellular drug via EV may not be the mode of action of this adaptive resistant line. Pretreatment of cancer cells with lansoprazole, a proton pump inhibitor (PPI) was reported to inhibit exosome production and subsequently caused higher cisplatin accumulation in human melanoma tumors [11]. PPI pretreatment was also found to enhance drug sensitivity and inhibit metastasis of gastric cancer cells [30]. The use of PPI in our study reduced EV secretion from both resistant OSCC lines. However, a small but significant difference in cisplatin sensitivity due to enhanced EV inhibition was only observed in H314 cells. We show that pretreatment of H314 cells with PPI resulted in a 10% reduction in cell viability compared to cells treated with cisplatin alone. While inhibition of EV production following PPI treatment in adaptive resistance cells H103/cisD2 did not apparently alter cisplatin sensitivity, as the cisplatin levels in EVs were notably less compared to those detected in culture medium. Taken together, the higher cisplatin content observed in H314-derived EVs and the significant effect of inhibiting EV production on the response of H314 to cisplatin indicates that the de novo resistant cells are at least partly depending on EVs to attenuate intracellular drug accumulation. In another word, OSCC cells that releases EVs containing cisplatin can be sensitized to the drug by inhibiting EV production.

## 4. Materials and Methods

### 4.1. Chemicals

3-(4,5-Dimethylthiazol-2-yl)-2,5-diphenyltetrazolium bromide (MTT) was purchased from Nacalai Tesque Inc (Kyoto, Japan). Cisplatin, Lansoprazole, sodium deoxycholate (SDC), chloroacetamide (CAA), formic acid and ethyl acetate were purchased from Sigma-Aldrich (St. Louis, MO, USA). DTT and trypsin were from BioRad (Hercules, CA, USA) and Promega (Madison, WI, Wisconsin) respectively. The cell culture media, DMEM nutrient mixture F-12 (DMEM-F12), were obtained from Invitrogen (Carlsbad, CA, USA). Antibodies used for western blotting include CD63, CD9, CD81, Na^+^/K^+^ ATP1B3, EGFR were from Santa Cruz Biotechnology Inc (Dallas, TX, USA), while antibodies against HSC70 and GAPDH were from Cell Signaling Technology (Denver, MA, USA) and Beta-actin from Sigma Aldrich (St. Louis, MO, USA).

### 4.2. Cell Lines and Maintenance

The OSCC cell lines used in this study (H103,H157,H314,H357, H376, H400, H413 and BICR31) have been described previously [13,14] and were cultured in complete media which consisted of DMEM/F12 (Sigma-Aldrich, St. Louis, MO, USA) supplemented with 10% FBS (Gibco, Life Technologies, Gaithersburg, MD, USA), 1% penicillin-streptomycin-glutamine (Gibco, Life-Technologies, Gaithersburg, MD, USA) and 0.5 µg/mL hydrocortisone (Sigma-Aldrich, St. Louis, MO, USA), in a humidified incubator maintained at 37 °C with 5% CO_2_.

### 4.3. Cell Viability Assay

OSCC cells were seeded in 96-well plates at a density of 5000 cells per well and allowed to attach for 18 h, and then treated with cisplatin at the indicated concentrations for 24 and 48 h. Cell viability was then measured using MTT assay. Briefly, media containing cisplatin were removed from the wells and replaced with complete media containing 0.5 mg/mL MTT. After 3 h, media were removed and the formazan crystals dissolved using DMSO. The absorbance was then measured at 570 nm using a Tecan Infinite 200 microplate reader. All cell viability assays were performed in quadruplicate and the percentage of viable cells was derived using the following formula:

Cell viability (%) = (Absorbance of treated group/Absorbance of control group) × 100%

To study exosome inhibition and cisplatin sensitivity, OSCC cells were pretreated with 25 µM of Lansoprazole, a proton pump inhibitor (PPI) for 24 h and followed by cisplatin for 24 h. The viability of OSCC cells after cisplatin treatment with and without PPI pretreatment was measured using MTT assay.

### 4.4. Establishment of Cisplatin-Resistant Cell Line

The cisplatin sensitive cell line, H103, was to establish a resistant subline using pulse treatment. Briefly, H103 cells were treated with 10 µM of cisplatin (IC_50_) for 24 h. The surviving cells were cultured in drug-free medium to recover and grow to confluency. Half of the confluent culture was then passaged to a new flask and treated again with 10 µM for 24 h and the surviving cells were then cultured in drug-free medium for recovery. After 2 weeks, the resistant colonies that survived were maintained in medium containing 10 µM of cisplatin. After 10 cell passages, the IC_50_ of the resistant cells was determined and compared to the parent cell control cells. Cells were maintained in drug-free medium for at least 4 weeks before experiments.

### 4.5. EV Isolation

OSCC cells cultured in the presence or absence of cisplatin (IC_50_) for 24 h were cultured in 40 mL of media containing EV-free FBS for 24 h. The cells were counted and the culture media centrifuged at 300× *g* for 10 min to remove floating cells, following by 2000× *g* for 10 min to remove dead cells at room temperature and 15,000× *g* for 30 min to remove cell debris at 4 °C. The supernatant was then centrifuged at 120,000× *g* for 80 min at 4 °C. The pellets from several tubes were combined and washed with phosphate-buffered saline (PBS) and pooled into one tube to concentrate the EVs by ultracentrifugation at 120,000× *g* for 80 min at 4 °C. The resulting EV pellet was resuspended in 100 µL of sterile PBS and stored at −80 °C. The protein content of the isolated EVs was determined using Bradford assays.

### 4.6. Transmission Electron Microscopy (TEM)

A 5 µL solution containing EVs was layered onto a formvar-coated 200 mesh copper-grid for 20 min. The copper-grid was washed with PBS briefly and fixed with 4% (v/v) glutaraldehyde for 1 min. The grid was then washed three times with deionized water and stained with 1% (w/v) uranyl acetate for 3 min. The grid was air-dried in the holder for 24 h. The images were obtained using a Hitachi transmission electron microscope model HT7700 (Tokyo, Japan) at a voltage of 100 kV.

### 4.7. Nanoparticle Tracking Analysis (NTA)

EV samples (~2 µg) were diluted with 500 µL of PBS and injected into the sample chamber of NanoSight NS300 (Malvern, UK) using a 1-mL sterile syringe until it fully occupied the sample chamber. Automatic settings for the maximum jump distance and a blur setting were used for the analysis. The detection threshold for the samples was 10 and a 30-s video recording the Brownian motion of the particles (EVs) for every sample was captured. A total of 3 biological replicates were analyzed for each sample. The captured videos were then analyzed using NTA software 3.0 (Malvern, UK).

### 4.8. Inductively-Coupled Plasma Mass Spectrometry (ICP-MS)

OSCC cells were pre-treated with cisplatin at the IC_50_ for 2 h followed by culture with drug-free medium containing EVs-free FBS. After 12 h, the culture medium was collected for EV isolation. Both cells and medium after removal of EVs were also collected for the following analysis. EVs and cells were treated with 20 µL and 100 µL of RIPA buffer respectively for 20 min on ice, vortex and digested with 100 µL and 200 µL of ~60% concentrated nitric acid at 70 °C for 2 h. The digested samples (lysed cells and EVs) and 200 µL of the culture medium depleted of EVs were diluted using deionized water to achieve a final volume of 8 mL. All samples were analyzed using Elan DRC-e ICP-mass spectrometer (Perkin-Elmer, Waltham, MA, USA) in the Institute of Tropical Agriculture, Universiti Putra Malaysia, Malaysia. A standard solution was prepared using Platinum ICP Standard in 7% HCL (Merck, Darmstadt, Germany). The sample matrix (mixture of RIPA buffer and nitric acid with no sample) was used as the negative control while the matrix containing cisplatin at 0.2 ng/mL, 1 ng/mL and 10 ng/mL were used as the positive controls. The concentration of platinum in cells was normalized based on cell numbers, while the reduction rate of cisplatin in the cells was measured using the following formula:

[(Intracellular platinum level in 2-h treatment) − (Intracellular platinum level in 12-h treatment)]/Intracellular platinum level in 2-h.

### 4.9. Protein Lysis and Digestion

EV samples for proteomic analysis were lysed in 50 µL of 1% (w/v) SDC dissolved in Tris pH 8.1 and boiled at 95 °C for 5 min, followed by sonication three times of 10 s each. The protein concentration was measured using Bradford assay. A total of 100 µg EV proteins was used for each sample. The proteins were denatured by 10 mM of DTT and incubated for 30 min at 50°C, followed by addition of 40 mM chloroacetamide for 20 min at room temperature in the dark. The samples were then digested using trypsin at a ratio of 1:100 and incubated on a shaker at 37 °C overnight. 1% formic acid was used to quench the digestion and the trypsinized sample was added with an equal volume of 100% water-saturated ethyl acetate, vortex thoroughly and centrifuged at 14,000× *g* for 5 min. The aqueous phase with peptides was transferred into a new tube and the ethyl acetate precipitation and peptide collection were repeated. The final collected peptides were dried in a vacuum concentrator.

### 4.10. Liquid Chromatography-Mass Spectrometry/Mass Spectrometry (LCMS/MS)

The proteomic analysis was carried out at Monash Biomedical Proteomics Facility, Monash, Australia. Data-dependent acquisition was performed on a QExactive™ Plus 1 mass spectrometer (Thermo Scientific, Waltham, MA, USA) coupled to a Dionex UltiMate^®^ 3000 RSLC nano Liquid Chromatography (LC) system (Thermo Scientific, Waltham, MA, USA) equipped with Acclaim PepMap RSLC analytical column (75 µm × 50 cm, nanoViper, C18, 2 µm, 100Å, Thermo Scientific, Waltham, MA, USA) and Acclaim PepMap 100 trap column (100 µm × 2 cm, nanoViper, C18, 5 µm, 100Å, Thermo Scientific, Waltham, MA, USA).

### 4.11. Protein Identification

The raw data files acquired from nano-LCMS were subjected into search engine Andromeda implemented in MaxQuant (v1.5.5.1) to identify the proteins with RS Uniprot human Swissprot iRT database and their respective label-free quantification values using in-house standard parameters. Principle component analysis (PCA) was conducted to evaluate the distribution of samples. The data were normalized based on the assumption that the majority of proteins do not change between the different conditions. Protein FDR cutoff was fixed at 1%. Statistical analysis was performed using Perseus after contaminants, “only identified by site” matches and reverse sequences were filtered out. The label-free quantification (LFQ) data was converted to log2 scale, samples were grouped by conditions and missing values were imputed based on normal distributions after all proteins. Proteins that had two or less valid values were eliminated. Protein fold-changes of the samples were calculated and their significance was determined using a two-sided *t*-test with error corrected *p*-values.

### 4.12. Protein Network Analysis

Proteins with differential expression between the EVs secreted by H103 (H103-EVs), H103/cisD2 (H103/cisD2-EVs) and H314 (H314-EVs) were compared in pairs: (i) H103-EVs and H103/cisD2-EVs, (ii) H103-EVs and H314-EVs, and (iii) H103/cisD2-EVs and H314-EVs. The protein lists were mapped onto the Vesiclespedia database in FunRich program to compare with the existing EV protein data available in Exocarta database (http://www.exocarta.org/). A Venn diagram was constructed to identify common proteins that were differentially expressed between cisplatin-sensitive and cisplatin-resistant OSCC-derived EVs (H103-EVs vs H103/cisD2-EVs, and H103-EVs vs H314-EVs). STRING (https://string-db.org/) was used for network analysis and functional annotation of the identified proteins. The functional annotation included Gene Ontology (GO) and Kyoto Encyclopedia of Genes and Genomes (KEGG) analysis.

### 4.13. Western Blot Analysis

OSCC cells and isolated EVs were lysed using RIPA buffer, and protein concentrations determined using Bradford assays. 20 µg of protein for each sample were resolved by SDS-PAGE and then transferred to nitrocellulose membranes. Next, the membranes were blocked with 5% (w/v) skimmed milk in Tris-buffered saline with 0.1% (v/v) Tween 20 (TBST) for 2 h and incubated with primary antibodies overnight at 4 °C. Membranes were then washed with TBST three times and then incubated with HRP-conjugated secondary antibodies for 2 h at room temperature. The blots were visualized using chemiluminescence detection reagents (Thermo Scientific, Waltham, MA, USA) and the resulting images captured using a C-DiGit Chemiluminescent Western Blot Scanner (LI-COR Bioscience, Lincoln, NE, USA) (Figure S4).

### 4.14. Statistical Analysis

Experiments were conducted with three replicates and data are expressed as means ± standard deviation (SD). The statistical analyses were carried out using Student’s t-test and one-way ANOVA with Tukey’s post hoc test. A *p*-value ˂ 0.05 was considered statistically significant.

## 5. Conclusions

In summary, these observations demonstrate the importance of EVs in the regulating the response of OSCC cells to chemotherapy. The data showed that EVs derived from drug resistant OSCC cells have high similarity in their protein profiles and the downregulation of proteins involved metal ion transportation could be regulators of drug resistance. In addition, we observed the use of inhibitor PPI could enhance the sensitivity of OSCC cells that release EVs with increased level of cisplatin, suggesting that inhibition of EV release could be a novel therapeutic approach to sensitize drug resistant OSCCs to chemotherapy.

## Figures and Tables

**Table 1:** Size distribution of oral squamous cell carcinoma (OSCC) extracellular vesicles (EVs) analyzed with Nanosight NS300.

EVs Derived from Cell Lines	Size of EVs (nm)					
	Mode	Mean	^a^ SD	^b^ D10	^c^ D50	^d^ D90
H103	103.6 ± 5.6	111.6 ± 17.9	46.1 ± 5.6	65.5 ± 5.7	97.1 ± 7.9	158.5 ± 22.2
H314	100.2 ± 12.5	130.0 ± 9.7	46.7 ± 11.4	78.4 ± 4.5	107.8 ± 4.3	179.7 ± 16.9
H103/cisD2	82.7 ± 22.5	99.8 ± 16.3	37.1 ± 1.0	55.0 ± 12.6	81.76 ± 19.8	132.4 ± 14.4

^a^ SD: standard deviation of particle sizes. ^b^ D10: less than 10% of particles are equals to or smaller than this size. ^c^ D50: less than 50% of particles are equals to or smaller than this size. ^d^ D90 less than 90% of particles are equals to or smaller than this size. EV: Extracellular vesicle.

**Table 2:** Growth rate and doubling time of OSCC cells.

Cell Line	Mean Growth Rate (h^−1^)	Doubling Time (h)
H103	0.0343	20.2 ± 3.9 ^a^
H314	0.0204	33.9 ± 1.4 ^b^
H103/cisD2	0.0259	26.9 ± 1.8 ^c^

Different letter indicates significant differences (*n* = 3, *p* < 0.05, Two-way Annova, Tukey’s post-hoc.).

**Table 3:** Fold changes of EV proteins involved in regulation of metal ion transportation (GO).

Proteins	^1^ Fold Change in EV		Function
	H314	H103/cisD2	
PACSIN3	−5.57688	−2.74937	Plays a role in endocytosis and regulates internalization of plasma membrane proteins
CTNNB1	−6.31522	−2.66626	Part of a complex of proteins that constitute adherens junctions
DLG1	−4.16456	−4.03571	May have a role in septate junction formation, signal transduction, cell proliferation, synaptogenesis and lymphocyte activation.
ATP1A1	−1.61594	−1.30331	Maintaining the electrochemical gradients of Na and K ions across the plasma membrane
ATP1B3	−1.87647	−1.651	Maintaining the electrochemical gradients of Na and K ions across the plasma membrane
CAB39	−2.64897	−1.95177	Component of a complex that binds and activates STK11/LKB1

^1^ Fold change compared to EV proteins of H103 cells.

## Supplementary Materials

The following are available online at https://www.mdpi.com/2072-6694/11/8/1166/s1, Figure S1: Bright field images of OSCC cells (magnification of 100×), Figure S2: Expression levels of EV marker proteins in OSCC-derived EVs, Figure S3: Concentration of platinum at 2 hours of cisplatin treatment and after 12 hours of cisplatin-free medium incubation, Figure S4: Western blotting. Table S1: List of genes with their proteins differentially regulated in EVs of cisplatin resistant OSCC, Table S2: Top 10 GO Cell component analysis, Table S3: Top 10 GO Biological processes analysis, Table S4: Top 10 KEGG Pathway analysis.
