# Supplementary material for: Cisplatin-Resistance in Oral Squamous Cell Carcinoma: Regulation by Tumor Cell-Derived Extracellular Vesicles
Source: Cancers (Basel). 2019 Aug 14;11(8):1166. doi: 10.3390/cancers11081166 (PMC6721547; doi:10.3390/cancers11081166)
Supplement: Supplementary file 1 [file cancers-11-01166-s001.pdf]

Supplemental Materials: Cisplatin-Resistance in Oral Squamous Cell Carcinoma: Regulation by Tumor Cell-Derived Extracellular Vesicles

Xin-Hui Khoo, Ian C Paterson, Bey-Hing Goh and Wai-Leng Lee

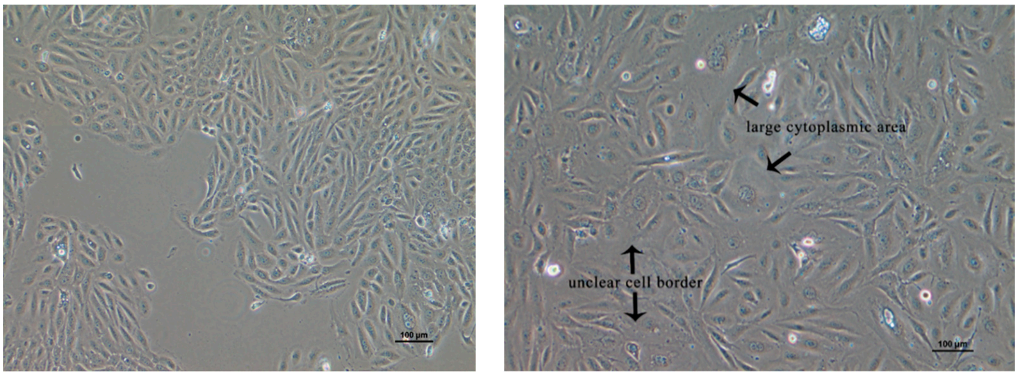

(**a**) (**b**)

Figure S1. Bright field images of OSCC cells (magnification of 100×). (**a**) H103 parental cell line, (**b**) H103/cisD2 cell line after 10 rounds of pulse treatment with cisplatin.

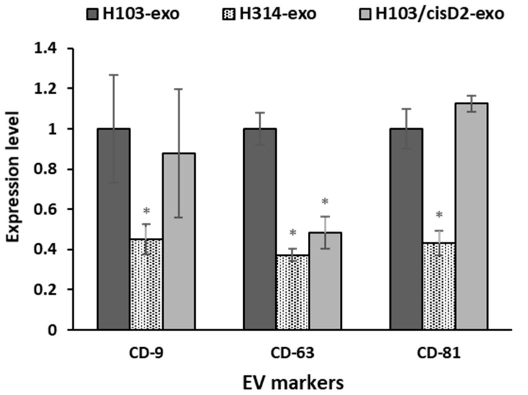

Figure S2. Expression levels of EV marker proteins in OSCC-derived EVs. Quantification of signal intensity was conducted using Image J. All the signals were normalized against intensity of HSC70. *indicated significant differences from EVs derived from H103 cells (H103-exo) ($n = 3$, $p < 0.05$, Two-way Annova, Tukey's post-hoc).

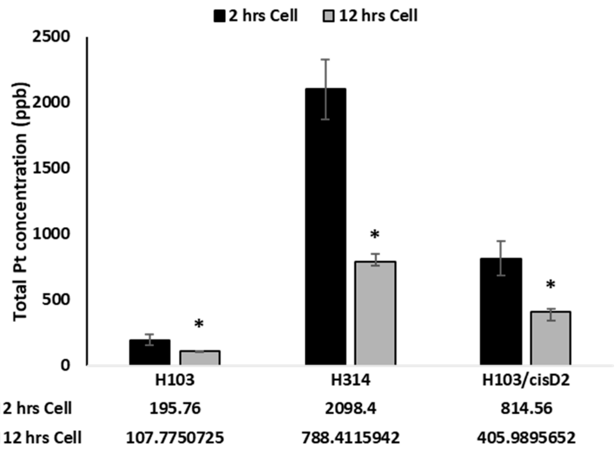

Figure S3. Concentration of platinum at 2 hours of cisplatin treatment and after 12 h of cisplatin-free medium incubation. * indicated significant differences from 2 h cells ($n = 3$, $p < 0.05$, Two-way Annova, Tukey's post-hoc).

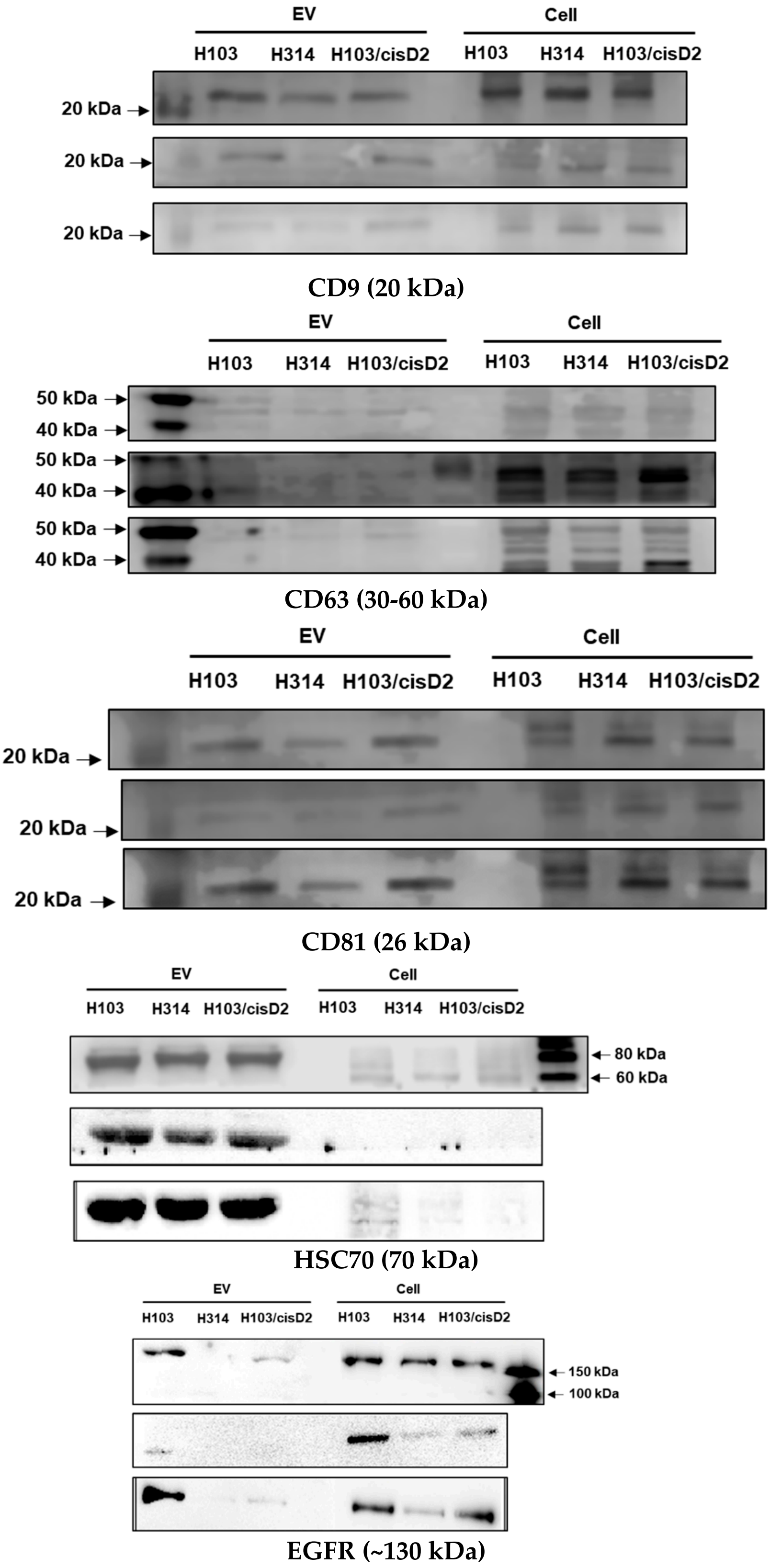

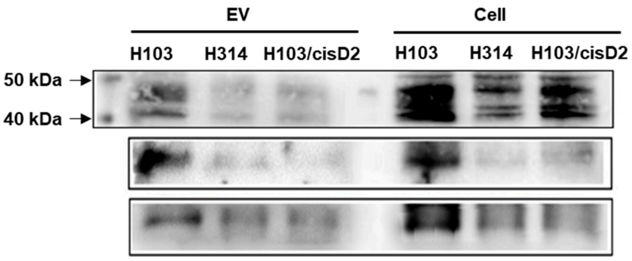

Figure S4. Western blotting (triplicates).

Table S1. List of genes with their proteins differentially regulated in EVs of cisplatin resistant OSCC.

Gene Name	Compare to H103-EVs, Fold Change of Protein in		Gene Name	Compare to H103-EVs, Fold Change of Protein in	
	H103/cisD2-EVs	H314-EVs		H103/cisD2-EVs	H314-EVs
CCT4	1.2213	1.31007	IGSF3	−3.32301	−3.37485
EDIL3	3.186	6.23853	IQGAP1	−0.822679	−1.40966
RPLP0	1.3014	1.23555	ITGA2	−1.81896	−1.40966
TGM2	1.90753	2.38332	ITGA3	−1.48316	−2.51412
ALCAM	−2.50111	−6.21093	ITGA6	−3.48457	−2.18924
ANXA1	−1.3593	−1.44066	ITGB4	−4.76825	−6.56137
ARRDC1	−3.10439	−2.82773	JUP	−2.05299	−4.65373
ATP1A1	−1.30331	−1.61594	LASP1	−1.31599	−1.55913
ATP1B3	−1.651	−1.87647	MARCKS	−1.74108	−2.19575
BASP1	−1.78838	−4.80193	MMP14	−3.05341	−3.35638
BSG	−1.49068	−2.30614	MPZL1	−3.16516	−3.79302
CAB39	−1.95177	−2.64897	MSN	−1.09643	−1.95576
CD44	−1.20358	−1.05904	MYO1B	−7.40723	−7.49534
CD59	−2.16691	−4.11755	MYOF	−4.39966	−3.68452
CD82	−4.1358	−4.50446	NCKAP1	−3.46425	−3.66785
CD9	−2.25156	−2.52558	NRAS	−1.1691	−1.6433
CDH3	−4.46503	−5.63997	PACSIN3	−2.74937	−5.57688
CFL1	−1.44838	−2.3573	PDCD10	−3.01543	−3.15708
CTNNA1	−2.69647	−7.3521	PKP3	−2.72952	−5.6661
CTNNA2	−2.56579	−4.83702	PLEC	−2.72199	−6.64841
CTNNB1	−2.66626	−6.31522	PLEK2	−1.62203	−2.48219
CTNND1	−2.82209	−6.38995	PLXNA1	−4.69861	−4.32371
CXADR	−3.50929	−1.98868	PROM2	−2.57304	−4.98848
DIP2B	−3.28048	−2.85467	PTGFRN	−2.95215	−8.19098
DLG1	−4.03571	−4.16456	RAB10	−0.568548	−0.5879
DOCK9	−2.25212	−2.04113	RDX	−2.31205	−2.36078
EGFR	−1.82431	−3.19202	RRAS	−2.42152	−2.89097
EHD2	−3.90278	−4.67087	S100A14	−4.80956	−5.97344
EHD4	−3.05061	−4.28404	S100A2	−4.96587	−3.68058
EPCAM	−5.16353	−6.23347	SCARB1	−2.09409	−2.42528
EPHA2	−3.1771	−5.6981	SLC16A1	−1.97213	−3.05675
EPS8L2	−1.38227	−2.56317	SLC1A5	−3.09421	−4.94067
EZR	−1.69251	−3.62617	SLC38A2	−3.77017	−3.3052
F3	−3.23329	−6.55877	SLC39A10	−3.48145	−3.56164
FSCN1	−1.05876	−1.78099	SLC3A2	−2.25988	−2.9735
GDI2	−1.16903	−2.67346	SLC7A5	−2.69542	−3.11446
GNAI3	−1.45609	−2.58369	TACSTD2	−6.49314	−7.4866
HLA−A	−1.92629	−1.23085	TPBG	−1.50519	−3.33372

Table S2. Top 10 GO Cell component analysis.

Pathway ID	Pathway Description	Count in Gene Set	False Discovery Rate
GO:0070062	Extracellular exosome	57	1.65e−32
GO:0070161	Anchoring junction	32	1.86e−31
GO:0005925	Focal adhesion	30	7.58e−31
GO:0030055	Cell-substrate junction	30	1.12e−30
GO:0005912	Adherens junction	31	1.22e−30
GO:0031988	Membrane-bounded vesicle	58	2.72e−29
GO:0030054	Cell junction	38	3.79e−27
GO:0044421	Extracellular region part	57	1.19e−26
GO:0005576	Extracellular region	57	9.61e−23
GO:0005886	Plasma membrane	54	3.35e−19

Table S3. Top 10 GO Biological processes analysis.

Pathway ID	Pathway Description	Count in Gene Set	False Discovery Rate
GO.0048870	Cell motility	25	1.15E−13
GO.0051674	Localization of cell	25	1.15E−13
GO.0009611	Response to wounding	24	1.29E−13
GO.0016477	Cell migration	24	1.29E−13
GO.0040011	Locomotion	28	1.89E−13
GO.0042060	Wound healing	22	1.90E−12
GO.0006928	Movement of cell or subcellular component	28	1.95E−12
GO.0007596	Blood coagulation	18	4.39E−10
GO.0032879	Regulation of localization	30	1.61E−08
GO.0007155	Cell adhesion	19	4.44E−07

Table S4. Top 10 KEGG Pathway analysis.

Pathway ID	Pathway Description	Count in Gene Set	False Discovery Rate	Matched Proteins
4810	Regulation of actin cytoskeleton	13	2.13E−10	CFL1,EGFR,EZR,IQGAP1,ITGA2,ITGA3,ITGA6,ITGB4,MSN,NCKAP1,NRAS,RDX,RRAS
5412	Arrhythmogenic right ventricular cardiomyopathy (ARVC)	8	3.95E−08	CTNNA1,CTNNA2,CTNNB1,ITGA2,ITGA3,ITGA6,ITGB4,JUP
5205	Proteoglycans in cancer	10	7.60E−07	CD44,CTNNB1,EGFR,EZR,IQGAP1,ITGA2,MSN,NRAS,RDX,RRAS
4520	Adherens junction	6	1.34E−05	CTNNA1,CTNNA2,CTNNB1,CTNND1,EGFR,IQGAP1
4670	Leukocyte transendothelial migration	7	1.34E−05	CTNNA1,CTNNA2,CTNNB1,CTNND1,EZR,GNAI3,MSN
4640	Hematopoietic cell lineage	6	3.57E−05	CD44,CD59,CD9,ITGA2,ITGA3,ITGA6
5206	MicroRNAs in cancer	7	4.13E−05	CD44,EGFR,EZR,FSCN1,MARCKS,NRAS,RDX
5213	Endometrial cancer	5	4.13E−05	CTNNA1,CTNNA2,CTNNB1,EGFR,NRAS
5200	Pathways in cancer	9	9.08E−05	CTNNA1,CTNNA2,CTNNB1,EGFR,ITGA2,ITGA3,ITGA6,JUP,NRAS
4530	Tight junction	6	0.000224	CTNNA1,CTNNA2,CTNNB1,GNAI3,NRAS,RRAS

© 2019 by the authors. Licensee MDPI, Basel, Switzerland. This article is an open access article distributed under the terms and conditions of the Creative Commons Attribution (CC BY) license (http://creativecommons.org/licenses/by/4.0/).
